# Proceedings of the Ohio State Dental Society

**Published:** 1883-12

**Authors:** 


### PROCEEDINGS.

### Proceedings of the Ohio State Dental Society.

The eighteenth annual meeting of the Ohio State Dental Society was held in the City Hall, Columbus, Wednesday, October 31st, and continued three days.

The meeting was called to order at 10:30, the President, J. W. Lyder, in the Chair.

The minutes of the last annual meeting were read.

The payment of dues was now in order, and the Treasurer found his coffers considerably replenished.

The names of W. H. Carson, D.D.S., of St. Clairsville, and L. D. Patton, D.D.S., of Defiance, were presented for memership, and, a ballot being had, they were declared elected.

After the disposition of some routine business the meeting adjourned.

### AFTERNOON SESSION.

The Society was called to order at 2 o'clock. The minutes of the morning session were read and approved.

Drs. D. Gale, French, and Templeton, of Pittsburg, were present as delegates from the Pennsylvania State Dental Society.

The Publication Committee reported that the transactions of last meeting had been published at an expense of $357.20. There has been received for the payment of this from advertising, etc., $306, leaving a balance of $46.70. There is, however, $15 uncollected, which will apply to the reduction of this balance.

The Committee on Ethics reported cases in which charges had been preferred, but as two members of the committee were absent, no action had been taken. The Chair filled the committee, which will formulate a report for a subsequent session of this meeting. Drs. Harrison and James are the new members.

The Committee on Voluntary Essays was filled by the appointment of Drs. Scott, of Defiance, Horton, of Cleveland, and Dr. F. H. Houghton, of Columbus.

"The Etiology of Dental Caries," was the first subject for discussion. It was opened by Dr. H. A. Smith. He proceeded to discuss the different characters of dental caries.

Dr. A. Berry followed, and joined issue with the former speaker, on the general assertion that vegetable food was more injurious to the health than animal. In fact, he thought that animal food should never go into the mouth.

During the discussion of this subject, Dr. Robinson read a paper of practical suggestions on the duty the patient owed to the dentist in the care of the teeth.

Dr. Whitney gave an interesting account of the affections of the gums that is very common among the inhabitants of the Sandwich Islands where he has practiced dentistry for many years. The disease there is similar to that found in this country, but is more common there than here.

Adjourned.

### EVENING SESSION.

The meeting was called to order at 7:30 o'clock. The proposition to change the by-laws so that the annual meeting should be held in the latter part of October, was taken up, and after consideration and due deliberation, the time for holding the annual meeting was fixed for the last Wednesday of October.

The regular subject for discussion—viz.: Etiology of Dental Caries, was now taken up.

Dr. J. Taft presented some thoughts upon the nature of decay, and upon the successive steps in the process of the affection. He called attention to the fact that, in decay, the bonds that hold together the constituents of the teeth must be broken; and then called attention to the nature of some of the agents that are able to do this, and their mode of action.

Dr. H. A. Smith remarked that, as sulphuric acid forms an insoluble compound, with the calcarious constituents of the teeth, is it not probable that sulphuric acid can be used in the treatment of the affection known as chemical abrasion; may it not be so applied as to form a protection to the teeth against the eroding agent?

Dr. Taft thought the idea was worthy of consideration. He suggested, however, that in the treatment, not only of chemical abrasion, but the ordinary decay as well, the general health condition, and the diet of the patient, should be taken into account; and it will often be found that a modification of these will result in much benefit to the teeth.

Dr. Smith suggested the application of nitrate of silver as a treatment for abrasion of the teeth, even though it might result in discoloring them for a time.

Dr. J. H. Warner said that, in regard to the application of nitrate of silver to sensitive dentine, it was not attended with pain.

Dr. French remarked that he had, in some instances, found intense pain result from the application of nitrate of silver to sensitive dentine.

Dr. Taft said that both gentlemen were correct to some extent. That nitrate of silver, applied to some cases of sensitive dentine, produced no appreciable pain, while in others intense and even prolonged pain is produced.

Dr. Whitney remarked that a large proportion of the dental troubles among the natives of the Sandwich Islands, had their beginning in chemical abrasion. He had also found that, among the Chinese, whose diet is almost wholly of rice, chemical abrasion is very common, and results very often in the loss of the teeth.

Adjourned.

### SECOND DAY.

The Society convened at 9:30, the President in the Chair.

The minutes of the last session read and approved.

The subject of "Dental Education," was now taken up, and an interesting paper on the subject was read by Dr. H. H. Harrison, of Cadiz.

The President called for an expression of the Society in regard to recommending students to certain dental colleges who have offered reduced rates to a certain number of students who shall be recommended by the respective State societies.

After considering the subject in its various aspects, the followlowing was passed unanimously:

"*Resolved*, That this Society hereafter declines to certify any student for a beneficiary scholarship in any dental college.

A letter was read from Dr. George Watt, in which he expressed regret for absence, and made valuable suggestions upon various points pertaining to the work of the Society in the future.

Drs. J. Taft and I. Williams were appointed a committee to consider and report upon the suggestions contained in Dr. Watt's letter.

Dr. Templeton, of Pittsburg, read an essay on the "Effect of Zymotic Diseases on the Dental Organisms," in which he took the ground that the effects of these diseases may be transmitted by heredity, and that such diseases as smallpox, scarlet fever, etc., affect the growth of permanent teeth in children.

The subject of Dental Education, was now resumed. Dr. A. Berry read a paper on the subject, after which it was discussed by Drs. Harrison, Smith, Warner, Waye, and others, all advocating a higher standard of educational attainments before entering the profession.

Adjourned.

---

SECOND DAY—AFTERNOON SESSION.

The Society met at 2:30 P. M.

The Committee on Ethics reported that, from the evidence presented, they were fully of the opinion that Drs. G. R. Warner and W. L. Gares, of Columbus, had violated the Code of Ethics, by the publication of an advertisement of a "Vitalized Air Apparatus."

On motion of Dr. W. P. Horton the report was referred back to the Committee for some emendation and correction of form.

The Committee, on Dr. Watt's suggestions, reported, recommending the appointment of two essayists and investigators for each subject of the programme for next meeting.

The recommendation was adopted.

The subject of Education was resumed.

Dr. J. Taft referred to the work accomplished in this direction by the associated boards of dental examiners, the initiation of which was effected at Lexington, Ky., in February last, when the representatives of the boards of nine States assembled, and proceeded with the preliminary steps for the formation of a permanent organization. A draft of a constitution, also the draft of a law in which were embodied all the requisite points for such a law were prepared. There was also prepared a list of questions upon the various branches of a dental curriculum as a guide for the various boards in their examinations. All of which were submitted to a meeting of boards assembled at Niagara Falls in August last, when the constitution was amended and adopted, and a permanent organization effected. The draft of a law, as prepared at Lexington, was thoroughly revised, and unanimously approved, and recommended to the various States, requiring amendments to their laws, or the passage of a law *de novo*.

The list of questions prepared at Lexington was then supposed to be a very fair one, and sufficiently stringent, if the various examing boards would keep their standards up to it. But the Association at Niagara, after carefully considering this list, recommended to the various boards throughout the country, that they raise their standards of examination twenty-five per cent. higher than that indicated by the list of questions just considered. Doubtless the influence of all this will be to induce young men preparing for the profession to go into the colleges and take a regular course. This will be the general influence of the *laws* regulating dental practice, and of the work of the boards of examiners as well, if they perform their duties uniformly properly.

Mr. Howard Hall, who has given special attention and study to the teeth of the various animals, and particularly to those of the horse, in respect to the various affections and diseases to which they are subject, was by invitation present, and made some interesting remarks upon the subject.

He remarked that he found the teeth of many horses so much affected as to seriously impair the work of mastication. The following affections he referred to as quite common, and which, in a more or less marked degree, interfered with the use of the teeth; viz., first, abrasion, or wearing away of the dentine between the vertical plates of enamel, thus making deep pits or cavities, and sharp, elongated prominences upon the molars, so as greatly to lessen their efficiency in grinding the food. Sometimes these cavities become quite sensitive. The proper method of treating such cases is to file off the projections till the tooth is of the proper form for efficient work.

Another serious affection is by the failure of a proper occlusion of the teeth upon one side. The jaws of a horse were exhibited in which the molars upon the left side slipped past each other about three inches, shear-fashion, when the teeth upon the right side were brought into apposition, they were, of course, very much elongated, and could not be put in apposition, and, of course, were entirely useless, and worse—they prevented the proper movement of the molars upon the right side. This horse starved to death because it could not masticate its food. The alveolus about the elongated teeth clearly showed that there had been severe disease of the gums.

Another difficulty often found is the prolonged retention of the temporary teeth, from which disease in the surrounding parts occurs, by which the use of the teeth is impaired or altogether prevented. The proper treatment in such cases is the removal of the offending tooth or teeth. Decay is often found in the teeth of the horse, and other animals as well. Sometimes the teeth of horses are so much decayed as to be useless, and, doubtless, they, in some cases, give great pain, and render mastication difficult. Attempts to fill cavities of decay in those teeth have been made, but the results in this direction have not been successful.

The teeth of herbivorous animals are more subject to decay than those of the carnivorous, as those of the latter seem to be of a firmer and more solid texture than those of the former; and the use to which they are subjected does not wear them as does that of the former.

There is very little, if any, probability of the eyes of the horse being affected by the ordinary affections of the teeth; this is, certainly, over-estimated in the popular mind.

Salivary calculus is often found on the teeth of the horse, and especially upon those not used. This, invariably, occasions disease of the gums.

Mr. Hall's remarks were received with marked attention and interest by the members of the Society. A hearty vote of thanks was extended to him.

The next subject, "Tin Foil as a Filling," was taken up. Dr. A. Berry was called to the Chair, when Dr. J. W. Lyder took up the subject and made some remarks on Robinson's Fibrous Tin, or filling material, for it is not wholly tin. He suggested that the admixture of a small portion of gold foil with the fibrous material would render a filling made with it more dense and resistant, and it would impart to the surface of the filling a better color. He suggested that teeth having periostitis or soreness of the contiguous parts could be filled with less pain to the patient with this material than with gold. A good filling for many cases, and especially for children's teeth, can be made by mixing the fibrous filling with the oxyphosphate or oxychloride filling. The method of preparing this filling is very simple. Take the requisite amount of fluid and mix the fibrous filling with it; then add the powder or oxide of zinc till the proper consistence is attained; then immediately introduce into the cavity.

Dr. A. Berry said that a filling made as just described by Dr. Lyder, is, for many cases, better than gold; and thinks it very valuable for dentists who do not use amalgams.

Dr. C. R. Butler: Tin foil is one of the first materials used for filling teeth, and notwithstanding this but few attain as good results from its use as are possible, or are easily obtained. He referred to the practice of Dr. Corydon Palmer, who attains the best results in the use of tin foil he had ever seen. Tin can and should be so refined as to have the cohesive property developed similar to that of gold.

In order to secure the best results the best tin foil should be obtained, of the best quality, perfectly pure, and carefully prepared. Gold and tin foil may be used in the same cavity, and in some cases with decided advantage.

In filling with tin, pluggers with larger points should be used than those for gold filling; both the mallet and hand-pressure is required in filling with tin.

I have found an adhesive wax very valuable in using rubber dam, for instance, to close little points of leakage, and to put upon conical teeth to retain the rubber in place, even when a clamp could not be used. This is accomplished by placing the rubber in proper position upon the tooth or teeth; then dry the surfaces of the teeth next to the rubber; then drop a little of the melted wax upon the tooth just in contact with the rubber; this will hold the rubber well in place, and without making undue pressure upon the gum.

The formula for the wax is: Gum Dammar, seven ounces; pure White Wax, four ounces; Canada Balsam, one drachm. These are melted and well mixed. This wax will be found to serve many good purposes.

Dr. D. G. French spoke in high commendation of the fibrous filling, but said it would not weld with gold; its efficient use, however, depends upon the skill of the operator. A bungler will make defective work with any and every material.

Dr. J. G. Templeton, of Pittsburg, was called upon, and said that the fibrous foil had almost superseded the use of amalgam as a filling in his practice. He had never seen an amalgam that would not shrink, although it is claimed that amalgams are manufactured that will not do so, and it was his opinion that nothing could be used better adapted to ruin a dentist's reputation than such a compound.

Dr. R. G. Warner remarked that the fibrous foil is a new thing, and not thoroughly tested, and should be used in general practice with care and caution.

Dr. Waye also gave some words of caution. He referred to some cases that seemed to indicate that, in the hands of the ordinary operator, better results could be obtained with good tin foil than with the fibrous filling. He could do nothing with the new material that he could not do just as well with a good quality of tin foil.

At this point the discussion was suspended.

Drs. Waye and Butler were appointed to fill vacancies in the Executive Committee.

Dr. Harrison, on behalf of the Committee on Ethics, made a statement in regard to the case of Drs. R. G. Warner and W. L. Gares, that, according to the rules of the Society, it would be necessary to notify Drs. Warner and Gares of the charges against them, and have them appear before the Committee with the persons preferring the charges.

Dr. Warner explained that his advertising consisted solely in the articles that appeared in the newspapers. The slips that had appeared he knew nothing about. They had been printed and distributed without his knowledge.

Dr. Horton remarked that he was interested in the matter only so far as to see justice done.

Adjourned.

EVENING SESSION.

The meeting was called to order at 7½ o'clock.

Dr. J. H. Siddall read a paper on "Old Engines and the New," after which he exhibited a hand piece and dental engine, which he had devised.

The election of officers for the ensuing year was now declared in order.

Dr. A. Berry, of Cincinnati, was elected President on the first ballot.

Dr. C. H. James, of Cincinnati, was elected Vice-President on the first ballot.

Dr. J. H. Warner was elected Secretary.

Dr. G. W. Keely was elected Treasurer.

The terms of Drs. J. Taft and F. H. Rehwinkel as members of the Board of Dental Examiners having expired they were re-elected for the term of three years.

Reference was made to the Dentists' Mutual Aid Association of Missouri, as a matter well worth the attention of the profession. Information on the subject; also the Constitution and By-Laws can be obtained of Dr. W. H. Sillito, of Xenia, Ohio. The publication of the transactions of this meeting was left in the hands of the Publication Committee.

The Committee on Ethics reported that the case of Drs. Warner and Gares, for violation of the Code of Ethics, would be presented in proper form for action, by the Society, to-morrow morning.

Dr. J. Taft described a method of constructing artificial crowns of teeth, devised and used by Dr. Mattison, of Chicago. The method embraces about all the good qualities of all the other methods in use, and is without some of their objectionable features. The appliance for constructing the crowns, which is very simple, was exhibited.

Dr. C. H. James was appointed a committee to revise the by-laws of the society.

The President elect was now installed.

A vote of thanks was extended to the outgoing officers.

Adjourned.

### THIRD DAY—MORNING SESSION.

The Society came to order—the minutes read and approved.

This morning Dr. W. P. Horton, of the Committee on Amendment of Dental Law, made the following report:

Your Committee on Amendment to the Dental Law would report to your honorable body, and recommend that the portion of the present law giving the appointing power of the Board to the

Ohio State Society be retained, and that the number of examiners be left to the discretion of the Society; that the law be so amended as to require a registration of all dentists in the State with the Clerk of the Court of Common Pleas in each county, and that a certified copy of said registration be sent by said clerk to the Chairman of said Board of Examiners; that the penalties for violations of the law be retained at about or nearly the same as the present.

A. F. Emminger,
W. P. Horton,
G. W. Keely.

The report was adopted.

The report of the Committee on Amendments to the Code of Ethics recommended the amendment of section 5, article 2, of the constitution by inserting the words "reprimand, suspension, or," before "expulsion." Also section 7 of the by-laws by striking out "the next regular meeting of," and adding to the section, the words "immediately thereafter."

Dr. Harroun of the Committee on Ethics reported as follows:

Your Committee on Ethics beg leave to report the following: Having examined the charges of unprofessional conduct in advertising, preferred against Drs. Warner and Gares of this city, find them to be the authors of quackish advertisements, as charged, which we deem a violation of the code of ethics of this society; and we hereby recommend that they be expelled or suspended from membership of this society, unless they make acknowledgement to the same.

C. H. Harroun,
C. H. James,
H. H. Harrison.

Dr. J. Taft moved that the Society proceed to take action on the report. The President said that the vote would be taken on highest penalty, expulsion, first; if that did not carry, then the next.

Dr. James said that there was not a man present who did not believe the code of ethics had been violated. The great question involved was the honor of the society and the profession.

Dr. Harrison, of Cadiz, said the law should be enforced unless the gentleman would retract and make amends. Dr. Taft took a similar position. He said the gentleman might at that time see wherein they erred, and he hoped they did, and were ready to make reparation.

Dr. I. Williams, of New Philadelphia, wanted to ask what the medical profession would do under similar circumstances if their code of ethics had been violated.

On the motion to adopt the report of the Committee on Ethics, the President stated that the question was then open for discussion, and that the accused could have an opportunity of stating their case.

Dr. R. G. Warner, on taking the floor, said that there seemed to be a determined effort to crush him; that the conclusion seemed to be foregone.

Prof. Smith moved that twenty minutes be granted Dr. Warner.

Dr. Warner then made his explanation, taking up the points in the objectionable advertisement *seriatim*. He was then questioned by Prof. Smith and Dr. James, and the latter then declared that Dr. Warner had convicted himself.

The vote was then taken on expulsion, but there was not a single aye.

Dr. Taft then moved that the vote be taken on suspending until satisfactory explanation and apology be made. This motion carried, and the President formally informed Drs. Warner and Gares of the action.

On motion of Dr. James, the time for making explanations and apology was deferred until the next annual meeting, at any time during the session, with the provision that in failure to make such satisfactorily, Drs. Warner and Gares be expelled from the Society.

Prof. Smith wanted to give an opportunity at the present meeting for apology, and Dr. Taft seconded the suggestion, and Dr. Gares said they had not thought that they had done wrong; but that since the Society had decided that such had been the case, they would promise not to advertise in that way any more. He was asked if he were sorry for what had been done, and replied that, if any wrong had been committed, they were sorry.

This apology was not accepted, however, and the motion of Dr. James remained in force.

The Executive Committee reported the following subjects for discussion at the next session, to be held in Columbus, on the last Wednesday, in October, 1884:

1. Improvements in Artificial Dentures.
2. Affections and Treatment of the Active Organs of Mastication.
3. Therapeutics in Dental Practice.
4. The Difficulties in Filling Teeth.
5. Pathological Conditions of the Mucous Membranes of the Oral Cavity; Causes and Appropriate Treatment.
6. Food, Digestion, and Nutrition, in Relation to the Healthful Condition of the Teeth and fluids of the Mouth.
7. Histology of Dental Tissues as Shown in the Processes of Development.

Dr. Siddall, of Oberlin, read a facetious production in rhyme of a miscellaneous nature, containing allusions, understood only by members of the Society, which was loudly applauded. [For which see this No. of the Register, page 592.—Ed.]

The President reported the appointment of the following committees:

*Executive Committee*—J. E. Robinson, Chairman, Columbus; C. R. Butler, Cleveland; C. J. Kelly, Hamilton.

*Committee on Ethics*—F. H. Rehwinkel, Chairman, Chillicothe; J. F. Siddall, Oberlin; C. H. James, Cincinnati.

*Committee on Membership*—W. H. Todd, Chairman, Columbus; J. R. Safford, Gallipolis; R. Gibbons, Warren.

*Committee on Voluntary Essays*—E. J. Waye, Chairman, Sandusky; J. W. Chance, London; Charles Welch, Wilmington.

*Committee on Publication*—J. H. Warner, Chairman, Columbus; I. Williams, New Philadelphia; G. W. Keely, Oxford.

*Committee on Amendment of Law*—W. P. Horton, Chairman, Cleveland; A. F. Emminger, Columbus; George W. Keely, Oxford.

Dr. F. H. Rehwinkel, Secretary of the State Board of Dental Examiners made his report, which states :

"The following candidates presented themselves for examination :

Tracy D. Rowley, Dexter City, Noble county ; Eugene D. Palchin, Shelby, Richland county ; Joseph W. Hisey, Massillon. Rowley and Patchin were examined and certificates of qualifications were issued to them. Mr. Hisey asked permission to withdraw from the examination after studying the questions submitted to him, without prejudice. He paid his fee and is entitled to examination next year."

The Board was reorganized as follows : Dr. J. Taft, President ; F. H. Rehwinkel, Secretary and Treasurer.

The Treasurer's report, as adopted, showed cash on hand, $245.50 ; expenses, $226.36 ; balance, $18.14.

Dr. Stroud, of Sandusky, was elected to membership.

At 12 o'clock the Society adjourned till the last Wednesday of Oct., 1884, at 10 A. M.

